# A multiscale computational model of spatially resolved calcium cycling in cardiac myocytes: from detailed cleft dynamics to the whole cell concentration profiles

**DOI:** 10.3389/fphys.2015.00255

**Published:** 2015-09-24

**Authors:** Janine Vierheller, Wilhelm Neubert, Martin Falcke, Stephen H. Gilbert, Nagaiah Chamakuri

**Affiliations:** ^1^Mathematical Cell Physiology, Max Delbrück Center for Molecular MedicineBerlin, Germany; ^2^Johann Radon Institute for Computational and Applied Mathematics (RICAM), Austrian Academy of SciencesLinz, Austria

**Keywords:** cardiomyocyte, dyad, calcium cycling, stochastic, spatially resolved cell model, FEM

## Abstract

Mathematical modeling of excitation-contraction coupling (ECC) in ventricular cardiac myocytes is a multiscale problem, and it is therefore difficult to develop spatially detailed simulation tools. ECC involves gradients on the length scale of 100 nm in dyadic spaces and concentration profiles along the 100 μm of the whole cell, as well as the sub-millisecond time scale of local concentration changes and the change of lumenal Ca^2+^ content within tens of seconds. Our concept for a multiscale mathematical model of Ca^2+^ -induced Ca^2+^ release (CICR) and whole cardiomyocyte electrophysiology incorporates stochastic simulation of individual LC- and RyR-channels, spatially detailed concentration dynamics in dyadic clefts, rabbit membrane potential dynamics, and a system of partial differential equations for myoplasmic and lumenal free Ca^2+^ and Ca^2+^-binding molecules in the bulk of the cell. We developed a novel computational approach to resolve the concentration gradients from dyadic space to cell level by using a quasistatic approximation within the dyad and finite element methods for integrating the partial differential equations. We show whole cell Ca^2+^-concentration profiles using three previously published RyR-channel Markov schemes.

## 1. Introduction

Cardiomyocyte muscle filament shortening and lengthening is a Ca^2+^ dependent process. The timing of contraction is controlled through electrical excitation via a process known as excitation-contraction-coupling (ECC). ECC is mediated through Ca^2+^, and is facilitated through an amplification process known as Ca^2+^-induced Ca^2+^ release (CICR). In ventricular myocytes, CICR is controlled locally by the colocalization of L-type Ca^2+^-channels (LCCs) in the T-tubule membrane on the one side of a dyadic cleft [aka Ca^2+^ release unit (CRU)] and ryanodine receptor channels (RyRs) in the junctional sarcoplasmic reticulum (jSR) membrane on the other side. Depolarization of the plasma membrane leads to the activation of LCCs, which causes Ca^2+^ entry from the extracellular space into the dyadic space. The influx of Ca^2+^ activates RyRs, which release Ca^2+^ from the sarcoplasmic reticulum (SR) (Fabiato and Fabiato, 1975). Within Ca^2+^ -release-units, Ca^2+^ dynamics are distinct from the myoplasm, with steep [Ca^2+^] gradients and several-fold higher [Ca^2+^] (Stern, 1992). The local release events (sparks) of the ~20,000 individual CRUs sum to produce a macroscopic whole-cell Ca^2+^ transient. The CRUs are coupled through myoplasmic Ca^2+^ diffusion, through SR Ca^2+^ diffusion, and through the spatially homogeneous membrane voltage.

Fundamental properties of ECC/CICR are: (i) that through a mechanism of local control of CICR, a graded LCC current produces a graded RyR Ca^2+^ release (Barcenas-Ruiz and Wier, 1987; Cannell et al., 1987; Stern, 1992); (ii) high whole cell CICR-gain at negative membrane potential V and lower whole cell CICR-gain at positive V (Altamirano and Bers, 2007); (iii) CICR is achieved by local Ca^2+^ release in highly localized cell-subcompartments, with dyadic space [Ca^2+^] rising to ~1000 × myoplasmic [Ca^2+^] (Koh et al., 2006) and strong gradients inside the dyads (Tanskanen et al., 2007; Hake and Lines, 2008; Hake et al., 2012); (iv) stochastic transition rates of LCCs depending on membrane potential (Cleemann et al., 1998) and small fluctuations in the number of LCCs can result in variability of AP duration and in early after depolarization formation (Tanskanen et al., 2005).

These properties illustrate that multiple length scales (tens of nanometers in the dyadic space to 100 μm cell size) and time scales (sub-millisecond for [Ca^2+^] changes in the dyad to tens of seconds for SR dynamics) are involved. To account for that multi-scale character, recently several models with spatially distributed Ca^2+^ release sites have been developed (Restrepo et al., 2008; Restrepo and Karma, 2009; Hatano et al., 2011; Williams et al., 2011; Nivala et al., 2012; Walker et al., 2014). These realistic models of CICR release aim at reproducing the fundamental properties of Ca^2+^ dynamics and at gaining independence from model-simplifying assumptions as far as possible with reasonable effort. Many of these models represent the dyadic space by a single compartment not resolving concentration gradients. Detailed spatially resolved models of the CRU have been developed which represent the steep local [Ca^2+^] gradients in the cleft (Koh et al., 2006; Schendel and Falcke, 2010; Hake et al., 2012; Schendel et al., 2012; Cannell et al., 2013; Stern et al., 2013 and others reviewed in Williams et al., 2010). However, a detailed CRU model has not yet been coupled to whole cell calcium dynamics and to whole cell electrophysiology, with a detailed representation of the spatial distributions of CRUs, challenges we address in this study.

We use mathematical multiscale techniques (Green Function, quasistatic approximation) to simulate a computationally efficient mathematical model of CRUs with spatially resolved [Ca^2+^] and stochastic state dynamics of all individual LC- and RyR-channels. The dynamics of up to 5120 CRUs is then embedded into simulations of the cellular concentration fields for Ca^2+^ and Ca^2+^-binding molecules as well as membrane potential time course.

## 2. Mathematical modeling

**Figure 1:**
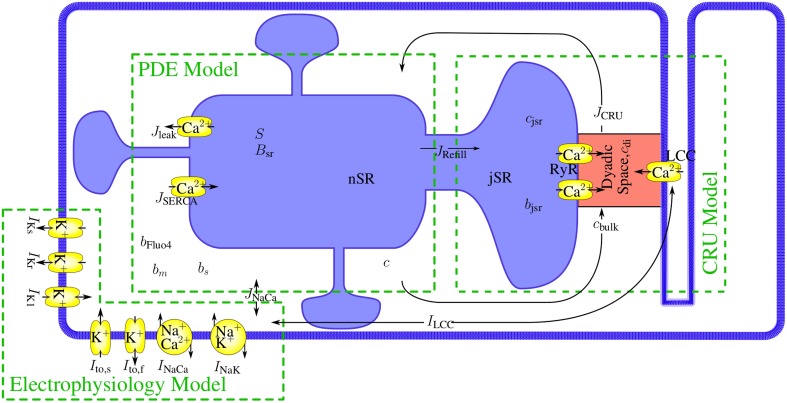
**The modules of the model and their interaction**. The mathematical model comprises a set of partial differential equations (PDEs) for the bulk concentrations of cytosolic and sarcoplasmic free Ca^2+^, cytosolic and sarcoplasmic mobile buffers and a cytosolic stationary buffer. The *N*_*c*_ Ca^2+^ release units (CRUs) are simulated all individually and are source terms in the bulk concentration dynamics PDEs. The state dynamics of each of their LC- or RyR-channels is a continuous time Markov chain. The concentration profile in the dyadic space is modeled in spatial detail with a quasistationary approximation, the dynamics of the concentrations of free Ca^2+^ and buffer in the jSR are determined by release into the cleft and refilling from the network SR (nSR). The electrophysiology model has been developed by Mahajan et al. (2008a). The LCC current in the CRUs and the Na^+^/Ca^2+^-exchanger flux couple the membrane potential dynamics directly to the concentration dynamics.

The mathematical model comprises a system of partial differential equations for the cytosolic and sarcoplasmic concentration dynamics, *N*_*c*_ models for the individual CRUs and a system of ordinary differential equations for the electrophysiology (see Figure 1). We present the individual modules first and then describe their coupling to a whole cell model. All parameter values are listed in Tables 1–5.

**Table 1:** **Buffering and diffusion parameters**.

**Parameter**	**Description**	**Value**
b${}_{m}^{tot}$	Total concentration of Troponin C (stationary buffer)	53.0 μM
b${}_{s}^{tot}$	Total concentration of Calmodulin (mobile buffer)	133.0 μM
B${}_{sr}^{tot}$	Total concentration of SR buffer	1500.0 μM
b${}_{Fluo-4}^{tot}$	Total concentration of Fluo-4	133.0 μM
k${}_{s}^{+}$	On rate for Troponin C binding	0.043 μM^−1^ ms^−1^
k${}_{s}^{-}$	Off rate for Troponin C binding	0.026 ms^−1^
k${}_{m}^{+}$	On rate for Calmodulin binding	0.8 μM^−1^ ms^−1^
k${}_{m}^{-}$	Off rate for Calmodulin binding	0.2 ms^−1^
k${}_{sr}^{+}$	On rate for SR buffer binding	0.1 μM^−1^ ms^−1^
k${}_{sr}^{-}$	Off rate for SR buffer binding	60.0 ms^−1^
k${}_{Fluo-4}^{+}$	On rate for Fluo-4 binding	0.0488 μM^−1^ ms^−1^
k${}_{Fluo-4}^{-}$	Off rate for Fluo-4 binding	0.0439 ms^−1^
D${}_{b}^{m}$	Diffusion constant of Troponin C	0.04 μm^2^/ms
D_*B*_	Diffusion constant of SR buffer	0.01 μm^2^/ms
D	Diffusion constant of calcium	0.22 μm^2^/ms
D_*S*_	Diffusion constant of SR calcium	0.20 μm^2^/ms
D_*Fluo*−4_	Diffusion constant of Fluo-4	0.033 μm^2^/ms
τ_*d*_	Refill-flux constant	0.5 ms
c_0_	Starting [Ca^2+^]_*i*_	0.275 μM

**Table 2:** **Exchanger and uptake parameters**.

**Parameter**	**Definition**	**Value**
f_*NaCa, high*_	Maximal factor of g_*NaCa*_ at CRU centers	2.5
f_*NaCa, low*_	Minimal factor of g_*NaCa*_ distant to CRUs	1.5
f_*NaCa, surf*_	Factor of g_*NaCa*_ at surface	0.5
K_*p*_	Uptake threshold	0.2 μM
Vp_*max*_	Strength of uptake	0.8 μM/ms
g_*NaCa*_	Strength of Na+/Ca^2+^-exchanger	0.84 μM/s
k_*sat*_	Constant	0.2
ξ	Constant	0.35
K_*m, Nai*_	Constant	12.3 mM
K_*m, Nao*_	Constant	87.5 mM
K_*m, Cai*_	Constant	0.0036 mM
K_*m, Cao*_	Constant	1.3 mM
c_*naca*_	Constant	0.3 μM

**Table 3:** **Physical constants and ionic concentrations**.

**Parameter**	**Definition**	**Value**
C_*m*_	Cell capacitance	3.1. × 10^−4^ μF
ν_*cell*_	Whole cell volume	2.58 × 10^−5^ μl
Ω	Simulated sub-cell volume	0.72 × 10^−5^ μl
x × y	cross-section of simulated volume	15 × 15 μm^2^
z	height of simulated volume	32 μm
F	Faraday constant	96.5 C/mmol
R	Universal gas constant	8.315 Jmol^−1^ K^−1^
T	Temperature	308 K
${\left[Na^{+}\right]}_{o}$	External sodium concentration	136 mM
${\left[K^{+}\right]}_{i}$	Internal potassium concentration	140 mM
${\left[K^{+}\right]}_{o}$	External potassium concentration	5.4 mM
*c*_*ext*_	External calcium concentration	1.8 mM
ν_*sr*_/ν_*cell*_	Ratio of SR to cell volume	0.1
ν_*jsr*_/ν_*cell*_	Ratio of jSR to cell volume	0.005
ν_*cyt*_/ν_*cell*_	Ratio of cytosolic volume to cell volume	0.895
κ	Constant in Equation (20)	1 nm^−1^
ϕ_0_	Constant in Equation (20)	−2.2

**Table 4:** **LCC and RyR parameters for (Stern et al., 1999; Cannell et al., 2013; Walker et al., 2014) model**.

**Parameter**	**Definition**	**Value**
N_*c*_	Number of CRUs	5120
N_*RyR*_	Average number of RyRs per CRU	50
N_*LCC*_	Average number of LCCs per CRU	12.5
r_*RyR, LCC*_	Ratio of RyRs and LCCs	4.0
**LCC PARAMETERS**		
g_*LCC*_	LCC single channel conductance	0.0546 μM^3^/ms
k${}_{p}^{0}$	Threshold for Ca-induced inactivation	90.0 μM
${\bar{c}}_{p}$	Threshold for Ca dependence of transition rate k_6_	60.0 μM
τ_*po*_	Time constant of activation	1 ms
r_1_	Opening rate	0.3 ms^−1^
r_2_	Closing rate	3 ms^−1^
s${}_{1}^{\prime }$	Inactivation rate	0.00195 ms^−1^
k${}_{1}^{\prime }$	Inactivation rate	0.00413 ms^−1^
k_2_	Inactivation rate	0.0001 ms^−1^
k${}_{21}^{\prime }$	Inactivation rate	0.00224 ms^−1^
T_*Ba*_	Time constant	450 ms
**RyR PARAMETERS Stern et al. (****1999****) AS MODIFIED BY Schendel and Falcke (****2010****)**		
g_*RyR*_	RyR permeability	2.33 μM^3^ s^−1^
k_*om*_	Activation rate	60 s^−1^
k_*im*_	Inactivation rate	5 s^−1^
k${}_{ac}^{max}$	Maximal activation rate	928.8 s^−1^
k${}_{in}^{max}$	Maximal inactivation rate	7.8 s^−1^
K_*jsr*_	Half max. value for *c*_*jsr*_-effect on RyR	550 μM
K_*ac*_	Activation threshold	8.5 μM
K_*in*_	Inactivation threshold	8.5 μM
λ	Asymmetric inactivation	27.5
**RyR PARAMETERS (Cannell et al., 2013)**		
g_*RyR*_	RyR permeability	2.33 μM^3^ s^−1^
k_*open*_	Opening rate	4.57 × 10^5^ *cdi*^2.12^ s^−1^
k${}_{open}^{max}$	Maximal opening rate	800 s^−1^
k_*close*_	Closing rate	245 *cdi*^−0.27^ s^−1^
**RyR PARAMETERS (Walker et al., 2014)**		
g_*RyR*_	RyR permeability	2.33 μM^3^ s^−1^
η	Ca^2+^ Hill coefficient	2.1
k_*open*_	Opening rate	k^+^ ϕ*cdi*^η^
k^+^	Opening rate constant	1.107 × 10^−4^ *ms*^−1^ μM ^−η^
k_*close*_	Closing rate	0.2 s^−1^
ϕ	${\left[Ca^{2+}\right]}_{jsr}$- dependent regulation term	$\phi_{b}+{\left({\left[Ca^{2+}\right]}_{jsr}/\phi_{k}\right)}^{4}$
ϕ_*k*_	${\left[Ca^{2+}\right]}_{jsr}$- dependent regulation affinity	1.5 mM
ϕ_*b*_	${\left[Ca^{2+}\right]}_{jsr}$- dependent regulation intercept	0.8025

*For the equations of the 4-state RyR model see (Stern et al., 1999; Schendel and Falcke, 2010)*.

**Table 5:** **Ionic current conductances**.

**Parameter**	**Definition**	**Value**
g_*Na*_	Peak *I*_*Na*_ conductance	12.0 mS/μF
g_*to, f*_	Peak *I*_*to, f*_ conductance	0.11 mS/μF
g_*to, s*_	Peak *I*_*to, s*_ conductance	0.04 mS/μF
g_*K*1_	Peak *I*_*K*1_ conductance	0.3 mS/μF
g_*Kr*_	Peak *I*_*Kr*_ conductance	0.0125 mS/μF
g_*Ks*_	Peak *I*_*Ks*_ conductance	0.1386 mS/μF
g_*NaK*_	Peak *I*_*NaK*_ conductance	1.5 mS/μF

### 2.1. PDE model

The dynamics of the cytosolic Ca^2+^ concentration, *c*, comprises of plasma membrane transport, release and uptake by the SR and binding to buffers. Plasma membrane transport is controlled by ion channels and the Na^+^/Ca^2+^-exchanger. The T-tubule network is an interface to extracellular fluid in the bulk of the cytosol due to which membrane fluxes like the Na^+^/Ca^2+^-exchanger contribute to bulk concentration dynamics (*J*_*NaCa*_). The Na^+^/Ca^2+^-exchanger flux through the plasma membrane ($J_{NaCa}^{pm}$) enters the partial differential equations as flux boundary condition. The flux *J*_*pump*_ describes the pumping of Ca^2+^ by SERCAs into the SR. A leak flux (often denoted by *J*_*leak*_) is not included as stochastic RyR openings during diastole account for SR leak. The Ca^2+^-binding molecules (*b*_*j*_, *j* = *s, m, f*) in the cytosol include stationary and mobile buffers and Fluo-4. The total concentration $b_{j}^{tot}$ is spatially homogeneous for all of the buffers. The partial differential equations for the cytosolic concentration fields and their boundary conditions are:

(1) $$\frac{\partial c}{\partial t}\;=\nabla \;\cdot \;(D\nabla c)+J_{cru}+J_{NaCa}-J_{pump}-J_{buf}$$

(2) $$\begin{array}{l} \;\frac{\partial b_{j}}{\partial t}=\nabla \;\cdot \;(D_{b}^{j}\nabla b_{j})+R_{j}\left(c,\;b_{j}\right),\;j=s,\;m,\;f\\ {\;\vec{n}\;\cdot \;D\nabla c|}_{\Gamma }=J_{NaCa}^{pm}\\ {\vec{n}\;\cdot \;D_{b}^{j}\nabla b_{j}|}_{\Gamma }=0,\;j=s,\;m,\;f, \end{array}$$

where **D** and ${\text{D}}_{b}^{j}$ are diagonal diffusion matrices. We denote the plasma membrane by Γ, and the cell volume by $\nu_{cell}\subset {\mathbb{R}}^{3}$. We simulate between 1 and 16 z-discs in a subvolume Ω < ν_*cell*_. The expressions for the fluxes are:

(3) $$J_{buf}\;=\sum_{j\;=\;1}^{N_{B}}R_{j}\left(c,\;b_{j}\right),\;R_{j}\left(c,\;b_{j}\right)\;=\;k_{j}^{+}\left(b_{j}^{tot}-b_{j}\right)c-k_{j}^{-}b_{j},\;j=s,\;m,\;f$$

(4) $$J_{cru}\;=\sum_{i\;=\;1}^{N_{c}}\frac{\nu_{cell}}{\nu_{cyt}}\frac{\Theta (R_{cru}^{i}-\left|\vec{r}-\vec{r_{i}}\right|)}{\frac{4}{3}\pi {\left(R_{cru}^{i}\right)}^{3}}\left[\sum_{j\;=\;1}^{N_{LCC}^{i}}I_{LCC}^{i,j}(t)+\sum_{j\;=\;1}^{N_{RyR}^{i}}I_{RyR}^{i,j}(t)\right]$$

(5) $$J_{pump}\;=\frac{V_{P}^{max}c^{2}}{K_{P}^{2}+c^{2}},$$

where Θ(*x*) denotes the step function

(6) $$\Theta (x)=\{\begin{array}{cc} 0 & x<0\\ 1 & x\geq 0 \end{array}$$

Ca^2+^ influx and release through LCC ($I_{LCC}^{i,j}(t)$) and RyR ($I_{RyR}^{i,j}(t)$) channels occurs in dyadic clefts. They are represented in the bulk dynamics by spherical source volumes with a radius determined by the numbers of LCCs ($N_{LCC}^{i}$) and RyRs ($N_{RyR}^{i}$) in the cleft. Their dependence on time t is caused by their stochastic behavior described in detail in Section 2.3. If an individual channel is open, its current obeys Equations (18 or 19), and is 0 otherwise.

We use the bidomain concept (Keener and Sneyd, 1998) for modeling cytosol and SR processes. It perceives both compartments to fill the same volume continuously with volume ratio ν_*cyt*_/ν_*cell*_ and ν_*sr*_/ν_*cell*_, resp. We include one buffer *B*_*sr*_ in the SR lumen. The partial differential equation for the SR Ca^2+^ concentration *S* and Ca^2+^ buffer *B*_*sr*_ are

(7) $$\begin{array}{l} \frac{\partial S}{\partial t}=\nabla \;\cdot \;(D_{S}\nabla S)-k_{sr}^{+}\left(B_{sr}^{tot}-B_{sr}\right)S+k_{sr}^{-}B_{sr}-J_{jsr}\\ \;+\frac{\nu_{cyt}}{\nu_{sr}}(J_{pump}) \end{array}$$

(8) $$\begin{array}{l} \;\frac{\partial B_{sr}}{\partial t}=\nabla \;\cdot \;(D_{B}\nabla B_{sr})+k_{sr}^{+}\left(B_{sr}^{tot}-B_{sr}\right)S-k_{sr}^{-}B_{sr}\\ {\vec{n}\;\cdot \;D_{S}\nabla S|}_{\Gamma }\;={\vec{n}\;\cdot \;D_{B}\nabla B_{sr}|}_{\Gamma }=0. \end{array}$$

**D**_*S*_ and **D**_*B*_ are diffusion matrices. Release appears as the flux *J*_*jsr*_ in the *S*-dynamics, which refills the individual junctional sarcoplasmic reticulae. Their concentration ($c_{jsr}^{i}$) dynamics are described in the context of the CRU models (Equation 21). The volume of the *i*th junctional SR is $\nu_{jsr}^{i}$. The refill flux is localized within a spherical volume with radius $R_{jsr}^{i}$ in the network SR:

(9) $$J_{jsr}\;=\sum_{i\;=\;1}^{N_{c}}\frac{\nu_{cell}\nu_{jsr}^{i}}{\nu_{sr}}\frac{\Theta (R_{jsr}^{i}-\left|\vec{r}-{\vec{r}}_{i}\right|)}{\frac{4}{3}\pi {\left(R_{jsr}^{i}\right)}^{3}}\;\frac{S({\vec{r}}_{i})-c_{jsr}^{i}}{\tau_{refill}}.$$

### 2.2. Electrophysiology model

In this subsection we briefly introduce the membrane potential model of the rabbit ventricular myocyte by Mahajan et al. (2008a). We assume that the membrane potential is uniform over the computational domain. The dynamics of the membrane potential *V* is described by:

(10) $$\frac{dV}{dt}\;=\;-\left(I_{ion}+I_{stim}\right).$$

*I*_*stim*_ is the stimulus current to depolarize the cell. *I*_*ion*_ comprises (Mahajan et al., 2008a):

(11) $$\begin{array}{l} I_{ion}=I_{Na}+I_{to,f}+I_{to,s}+I_{Kr}+I_{Ks}+I_{K1}+I_{NaK}\\ \;+\;I_{CaL}+I_{NaCa}. \end{array}$$

I_*Na*_ is the fast Na^+^ current, I_*K*1_ is the inward rectifier current, I_*to, f*_ is the fast component of the rapid outward K^+^ current, I_*to, s*_ is the slow component of the rapid outward K^+^ current, I_*Kr*_ is the rapid component of the delayed rectifier current, I_*Ks*_ is the slow component of the delayed rectifier current and I_*NaK*_ is Na^+^/K^+^ pump current. These currents and the equations defining them are described in detail in Mahajan et al. (2008a). The Na^+^/Ca^2+^-exchanger current *I*_*NaCa*_ is determined by the spatial integral of the corresponding fluxes in the cytosolic concentration dynamics

(12) $$I_{NaCa}=\frac{\nu_{cyt}F}{\nu_{cell}C_{m}}\left(\frac{\nu_{cell}}{\Omega }\int_{\Omega }J_{NaCa}d\Omega +\frac{A_{cell}}{\Gamma }\int_{\Gamma }J_{NaCa}^{pm}\;d\Gamma \right).$$

A prefactor *f*_*NaCa*_ for each finite element has been introduced to simulate the distribution of the Na^+^/Ca^2+^-exchanger according to Jayasinghe et al. (2009). The LCC-current *I*_*CaL*_ is the sum of all individual single channel currents (Equation 19) from the CRU models

(13) $$I_{CaL}\;=-2\alpha \sum_{i\;=\;1}^{N_{c}}\sum_{j\;=\;1}^{N_{LCC}^{i}}I_{LCC}^{i,j}.$$

The factor α = *Fν*_*cell*_/*C*_*m*_Ω converts the current in terms of ions/s into *p*A/*p*F and at the same time scales the currents up from values obtained with the simulation volume Ω, which is set by the number of simulated z-discs, to values corresponding to a realistic cell volume ν_*cell*_. Similarly, the factor *A*_*cell*_/Γ in Equation (12) scales the plasma membrane component from simulated (Γ) to whole cell area (*A*_*cell*_). *C*_*m*_ is the cell membrane capacitance and *F* the Faraday constant.

### 2.3. The CRU model

**Figure 2:**
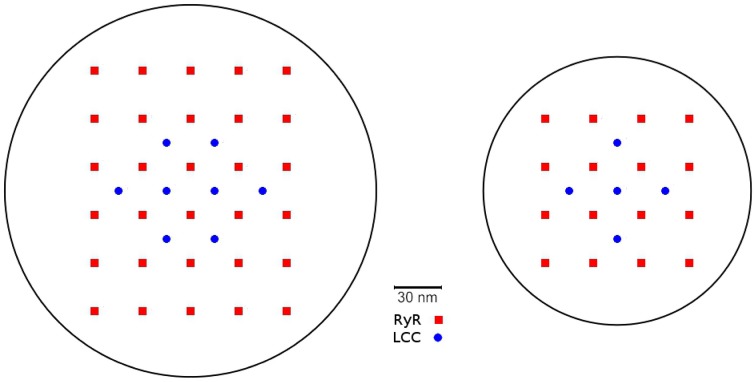
**Two examples for channel placement in the CRU model**. For 30 RyRs and 8 LCCs (left) and 16 RyRs and 5 LCCs (right). The number of RyR channels per CRU obeys an exponential distribution with an average of 50 across all CRUs.

The CRU model was based on a model previously developed by Schendel et al. (2012). We adapted it in a few key points to properly interact with the PDE model described above. We omit the index numbering the CRUs in this section for simpler notation. The dyadic space is modeled as a cylinder of variable radius and a height of 15 nm containing LCCs and RyRs, which are placed in regular arrays at the bottom and top of the cylinder (see Figure 2). The number of RyRs in each CRU was randomly determined using an exponential distribution with mean 50. For each four RyRs there was one LCC. The diameter of the cylinder was determined for each CRU such that there was a margin of 60 nm between the outermost channel and the boundary of the cleft.

#### 2.3.1. Stochastic channel gating

**Figure 3:**
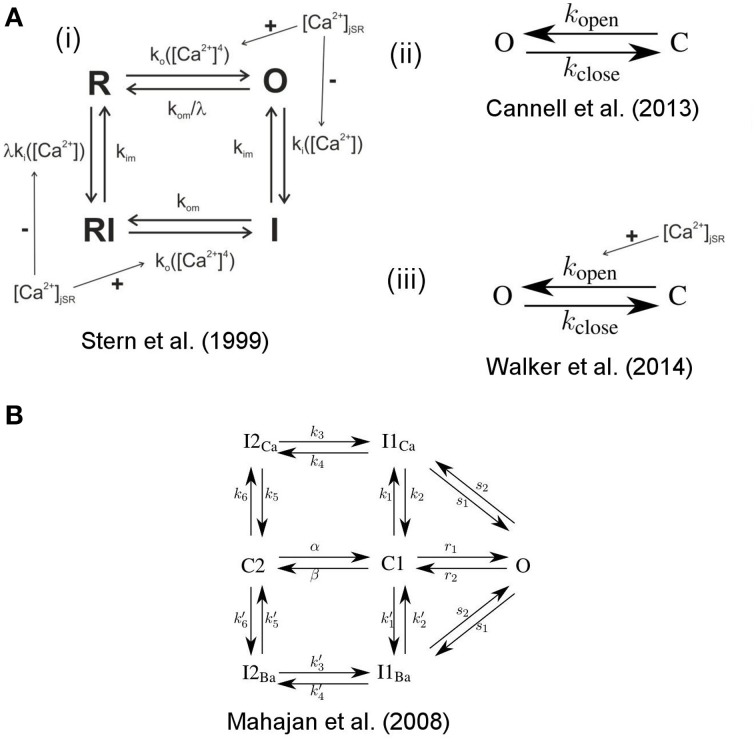
**State scheme for the RyR and LCC Markov Models**. **A(i)** 4-state RyR-channel model of Stern et al. (1999), O denotes the open, I the inactivated, and R the resting state. The activation rate k_*o*_ is a fourth order Hill function of dyadic Ca^2+^, the inactivation rate k_*i*_ is a first order Hill function of Ca^2+^. The Ca^2+^ dependent rates are influenced by the Ca^2+^ concentration in the jSR. The model was originally developed by Stern et al. (1999) and was modified by Schendel et al. (2012). **A(ii)** 2-state RyR-channel model of Cannell et al. (2013) where O denotes the open and C the closed state and *k*_open_ and *k*_close_ are polynomial functions of [Ca^2+^]. **A(iii)** 2-state RyR-channel model of Walker et al. (2014) where O denotes the open and C the closed state, *k*_open_ is a polynomial function of both [Ca^2+^] and [Ca^2+^] _jsr_ (i.e., *k*_open_ is influenced by the Ca^2+^concentration in the jSR) and *k*_close_ is a constant. **(B)** 7-state scheme for the LCC according to Mahajan et al. (2008a) with the states O open, I1_*Ca*_, I2_*Ca*_ Ca^2+^ dependent inactivated states, C1, C2 closed states, I1_*Ba*_, I2_*Ba*_ Ca^2+^ independent inactivated states (for details see Mahajan et al., 2008a).

The state dynamics of the LC- and RyR-channels are simulated with Markov models. For the RyRs we explored three models, the first is a model originally developed by Stern et al. (1999) in it's modified version by Schendel et al. (2012). It is a four state model [see Figure 3A(i)], including one open, one resting and two inactivated states. It features RyR inhibition in case of high cytosolic or low jSR Ca^2+^concentrations. For an in-depth description of this model see (Schendel et al., 2012). The second model is the two-state model [see Figure 3A(ii)] developed by Cannell et al. (2013), in which termination of CICR is mediated through the steep Ca^2+^dependence of the RyR closed time. With a small decline in jSR [Ca^2+^] the Ca^2+^flux via open RyRs declines, causing a decline in local dyadic [Ca^2+^], which in turn causes a decrease in the open probability of neighboring RyRs, a process known as induction decay. This RyR model does not rely on experimentally un-substantiated biophysical mechanisms for CICR termination, such as dyadic/cytoplasmic RyR Ca^2+^-dependent inactivation, or RyR-lumenal Ca^2+^-dependent inactivation. The third model is the two-state model [see Figure 3A(iii)] developed by Walker et al. (2014) (adapted from Williams et al., 2011) which incorporates modulation of the RyR-opening rate by junctional RyR-lumenal [Ca^2+^] ($c_{jsr}^{i}$). This model has a fixed closing rate, and, in accordance with the experimental data of Cannell et al. (2013), there is only weak regulation of the RyR opening-rate when ($c_{jsr}^{i}$) is < 1 mM. It is of note that the opening rate of this last RyR-Model is theoretically unbound, however in our simulations opening rates larger than 0.7 ms^−1^ were not encountered.

For the LCCs we used the 7-state model developed by Mahajan et al. (2008a) (see Figure 3B) which exhibits both Ca^2+^ dependent and Ca^2+^ independent inactivation.

Each channel's state was chosen randomly according to the steady state distribution for the initial values. The transition times between different states were determined using the Gillespie Algorithm for time dependent transition rates. That algorithm determines the time of an event as the time of the crossing of a random threshold by an integrated propensity for each individual Markov chain. With the given rate of changes in membrane potential and Ca^2+^ concentration, propensities are strongly time dependent. Hence, before we finally accept a calculated update of *V* and *c*, we verify that we have taken into account all events triggered by propensity crossings by the update during the iteration step. The precise definition of the transition rates are given in Schendel et al. (2012), Cannell et al. (2013), and Walker et al. (2014) for the three RyR-Models and in Mahajan et al. (2008a) and the accompanying CellML data (Mahajan et al., 2008b) for the LCC Model (there are slight discrepancies in the equations between the CellML data and the original paper; the equations we use are from the CellML data). In some cases we had to adjust the constants involved in the equations, and these are given in Table 4.

The frequency of transitions becomes very large with a large number of CRUs and with many channels in each CRU. While this in itself does not have a significant impact on the speed of the stochastic algorithm, it can force very small timesteps on the PDE model. In order to alleviate this problem and allow for longer timesteps we take advantage of the properties of the stochastic process. Those channel state transitions which do not represent either openings or closings are (within a given iteration step) independent stochastic events. We can therefore execute an arbitrary number of such transitions in a single iteration. This approach scales almost independently of the total number of states of the channel model. It is only dependent on the frequency of transitions from open to closed state or vice versa.

#### 2.3.2. Ca^2+^profile in the CRU

The Ca^2+^concentration in the cleft (*c_di_*) is modeled by the following equation in cylindrical coordinates:

(14) $$\begin{array}{l} \frac{\partial c_{di}}{\partial t}=\sum_{i\;=\;1}^{N_{LCC}}J_{\text{LCC}}^{i}+\sum_{j\;=\;1}^{N_{RyR}}J_{\text{RyR}}^{j}+D_{c}\Delta_{r,\varphi }c_{di}(\vec{r})-\frac{\partial }{\partial z}J_{z}. \end{array}$$

Because the time to reach the stationary concentration profile upon opening or closing of a channel is about one order of magnitude shorter than the timescale of channel state transitions, we assume steady state for *c_di_*. We make a separation ansatz (yielding only a negligible error as discussed in Schendel and Falcke, 2010):

$$c_{di}(r,\;\varphi ,\;z)=f(r,\;\varphi )\;\cdot \;Z(z).$$

The large aspect ratio radius/height of the dyadic space renders gradients in the *z*-direction of the cylinder due to channel fluxes negligible (Schendel et al., 2012). However, electro-diffusion due to differential charge densities on T-tubule and jSR membrane causes a substantial gradient. Soeller and Cannell (1997) determined the charge density profile shaping *Z*(*z*) (Soeller and Cannell, 1997). With their result we obtain

$$Z(z)=e^{-2\phi_{0}e^{-\kappa z}}.$$

The values of ϕ_0_ and κ are listed in Table 3. To find *f* we consider (Equation 14) for a given configuration of open channels. The flux through the channels is constant due to the steady state assumption. We describe the channel fluxes as Dirac-δ-functions in space with the channel currents as pre-factors, integrate the equation over *z* and obtain

(15) $$\begin{array}{l} \Delta_{r,\;\varphi }f(r,\;\varphi )=-\frac{1}{D_{c}h^{*}}[\sum_{i\;=\;1}^{N_{LCC}}I_{LCC}^{i}\delta (r-r_{i},\;\varphi -\varphi_{i})\\ \;+\sum_{j\;=\;1}^{N_{RyR}}I_{RyR}^{j}\delta (r-r_{j},\;\varphi -\varphi_{j})]\text{​}, \end{array}$$

where $h^{*}=\int_{0}^{h}Z\left(z\right)dz$^1^. Solving this using the Green function *G*((*r*, φ), (*r*_*i*_, φ_*i*_)) we find

(16) $$\begin{array}{l} f(r,\;\varphi )-c_{bulk}=\frac{1}{h^{*}D_{c}}\sum_{i\;=\;1}^{N_{LCC}}I_{LCC}^{i}\;G((r,\;\varphi ),\;(r_{i},\;\varphi_{i}))\\ \;+\sum_{j\;=\;1}^{N_{RyR}}I_{RyR}^{j}\;G((r,\;\varphi ),\;(r_{j},\;\varphi_{j}))\\ \;G(r,\;r_{i})=\frac{1}{2\pi }\log\left(\frac{∥\hat{r_{i}}-r∥}{q∥r_{i}-r∥}\right)\text{ for }r_{i}\neq \;0\\ \;G(r,0)=\frac{1}{2\pi }\log\left(\frac{R}{∥r∥}\right) \end{array}$$

where *R* is the radius of the dyadic space, $q=\frac{R}{∥r_{i}∥}$, and $\hat{{\text{r}}_{i}}=q^{2}{\text{r}}_{i}$. *c*_*bulk*_ is the Ca^2+^concentration at the boundary of the cleft (cylinder barrel) as computed by the PDE model.

With

$$\eta (\vec{r_{i}},\;\vec{r})=\frac{Z(z)}{h^{*}D_{c}}G((r,\;\varphi ),\;(r_{i},\;\varphi_{i}))$$

*c_di_* can be written as

(17) $$c_{di}(\vec{r})=Z(z_{i})c_{bulk}+\sum_{i\;=\;1}^{N_{LCC}}I_{LCC}^{i}\;\eta (\vec{r_{i}},\;\vec{r})+\sum_{i\;=\;1}^{N_{RyR}}I_{RyR}^{i}\;\eta (\vec{r_{i}},\;\vec{r}).$$

The currents $I_{LCC}^{i}$ and $I_{RyR}^{i}$ depend themselves on the local Ca^2+^ concentration at the channel mouth:

(18) $$I_{RyR}^{i}=g(c_{jsr}-\;c_{di}({\vec{r}}_{i}))\text{ for }i\in N_{\text{RyR}}$$

(19) $$I_{LCC}^{i}=J_{L}\delta V\frac{c_{\text{ext}}e^{-\delta V}-\;c_{di}({\vec{r}}_{i})}{1-e^{-\delta V}}\text{ for }i\in N_{LCC}$$

with δ = 2*F*/(*RT*). Here, ${\vec{r}}_{i}$ denotes the location of the mouth of the *i*th channel. Inserting Equations (18, 19) into Equation (17) and evaluating it at each channel mouth defines a system of linear equations for the concentration values setting the currents for given values of *c*_*jsr*_ and *c*_*bulk*_. The coefficients of that system can be calculated in advance of a simulation from the cleft geometry, which renders the simulation very efficient.

#### 2.3.3. Buffers in the dyadic space

In order to employ the two state RyR-models by Cannell et al. (2013) and Walker et al. (2014) (see the paragraph on *Stochastic Channel Gating* below) we found it necessary to introduce a form of Ca^2+^buffering in the dyadic space. This is to be expected since in the original papers the models were fitted to data from experiments conducted in the presence of buffers such as Calmodulin, Fluo-4, and ATP. Cannell et al. (2013) used also in their simulations high buffer concentrations within the dyadic space, and in order to adapt our model to this degree of buffering we introduce a linear buffer factor β. The coupling factor η now takes the form:

$$\eta (\vec{r_{i}},\vec{r})=\frac{1}{\beta }\frac{Z(z)}{h^{*}D_{c}}G((r,\varphi ),(r_{i},\varphi_{i}))$$

We adjusted β so that the Ca^2+^ concentration at the channel mouths and the average dyadic Ca^2+^ concentrations matched the values given in the paper by Cannell et al. (2013). An approximation of [Ca^2+^]_*i*_ that would be measured by a single-wavelength Fluo-4 experimental recording (denoted ${\left[Ca^{2+}\right]}_{i}^{exp}$) was calculated from the Ca^2+^-bound Fluo-4. The *in vitro* calibration approach described in Takahashi et al. (1999) was used:

(20) $${\left[Ca^{2+}\right]}_{i}^{exp}=K_{d}\times (F-F_{min})/(F_{max}-F),$$

where *K*_*d*_ is the dissociation constant of Fluo-4, *F* is the experimentally measured fluorescence intensity (here the [Ca^2+^-bound Fluo-4]), *F*_*max*_ is the measured fluorescence intensity in Ca^2+^-saturated dye (here this is set as the maximum [Ca^2+^-bound Fluo-4], i.e., $b_{Fluo-4}^{tot}$), and *F*_*min*_ is the measured fluorescence intensity in the absence of Ca^2+^ (here set to zero) (see Table 1).

#### 2.3.4. Modeling jSR dynamics

Each dyadic space is paired with its own junctional SR (jSR) compartment. We assume spatially uniform Ca^2+^concentration in the jSR. Ca^2+^dynamics in the jSR depends on the release flux through the RyRs and a refill flux from the network SR (nSR), which we assume to depend simply on the concentration difference *S* − *c*_*jsr*_. The buffering by Calsequestrin is modeled using the fast buffer approximation.

(21) $$\begin{array}{l} \frac{dc_{jsr}}{dt}=\beta_{jsr}\left(\frac{S(\vec{r})-c_{jsr}}{\tau_{refill}}-\frac{1}{\nu_{jsr}}\sum_{j\;=\;1}^{N_{RyR}}I_{RyR}^{j}\right)\\ \beta_{jsr}={\left(1+\frac{nK_{csqn}B_{csqn}}{{\left(K_{csqn}+c_{jsr}\right)}^{2}}\right)}^{-1} \end{array}$$

$S\left(\vec{r}\right)$ denotes the free nSR Ca^2+^concentration at the location $\vec{r}$ of the spherical volume representing refilling in the *S*-dynamics (Equation 7). This equation is solved numerically by linearization with very small time steps (< 10^−4^ ms).

### 2.4. Module interactions

The modules interact on a time scale longer than a single iteration step by the dependencies of the dynamics on Ca^2+^, *V*, and other state variables. Instantaneous interactions are important for the algorithmic realization of the time integration. We use for the completely coupled system an Euler forward method. The instantaneous interactions are between the PDE system and the CRU models mediated by Ca^2+^ currents and concentrations, between the CRU models and the electrophysiological model by the LCC current, and between the PDE system and the electrophysiological model via the bulk and plasma membrane components of the Na+/Ca^2+^-exchanger flux (see also Figure 1).

#### 2.4.1. Bulk concentrations—electrophysiology

We use the Ca^2+^ concentration spatially resolved at the plasma membrane for calculating local values of $J_{NaCa}^{pm}$ and analogously in the bulk for *J*_*NaCa*_, and then average (Equation 12) to obtain the current entering the membrane potential dynamics. Vice versa, $J_{NaCa}^{pm}$ serves as boundary condition and *J*_*NaCa*_ as bulk source term for the PDEs.

#### 2.4.2. Bulk concentrations—CRUs

The value of the Ca^2+^ concentration *c* determined by the PDEs is averaged along the rim of a cleft volume and then serves as boundary condition *c*_*bulk*_ in Equation (17) to determine the RyR and LCC currents ($I_{LCC}^{i,j}$ and $I_{RyR}^{i,j}$) in each CRU. Vice versa, these currents determine the source terms for the bulk concentration dynamics in Equation (1).

#### 2.4.3. Electrophysiology—CRUs

The membrane potential affects the LCC currents (Equation 19) and vice versa the sum of all individual LCC currents the membrane potential (Equation 13).

### 2.5. Numerical approach

We used a piecewise bi-linear finite element method for the solution of the spatially three-dimensional reaction-diffusion model including the complex distribution of CRUs at multiple z-discs. The first challenge was the fine scale resolution of the computational grid to resolve the strong concentration gradients at the boundary of the CRUs. We take the equidistant tetrahedral elements with the size of 0.05 μM in our computations. The next challenge is to deal with the adaptive time stepping schemes for solving the reaction-diffusion systems. Due to the fast transitions of the channel opening/closings in a CRU, the time scales vary from tens of microseconds to milliseconds. To resolve such rapid changes adaptive and higher order time steppings are inevitable to treat the very smooth diffusion effects as efficiently as possible. To this end, we use higher order linearly implicit Runge-Kutta methods for time discretization of reaction-diffusion systems, see (Lang, 2001; Chamakuri et al., 2012). These belong to a large class of methods which try to avoid the nonlinear system and replace it by a sequence of linear ones. Also, this allows the use of adaptive timescales in the simulations. Specifically, we employed a second order Rosenbrock method called ROWDA (Lang, 2001), and we avoid the time discretization implications of this problem as described in Chamakuri and Rüdiger (2012, Section 4.2).

Our parallel implementation of the discretization routines are based on the public domain package DUNE (Bastian et al., 2008), especially the *dune-pdelab* discretization module. The parallel linear solvers depend on the *dune-istl* module. Based on this, we developed a finite element simulator to solve the whole cell calcium cycling model. The internal parallel Cartesian (called Yasp) grid in DUNE is used for the parallel grid constructions. In our domain decomposition approach, the original domain is partitioned into subdomains and each subdomain is assigned to a single processor. In our computations, we used a non-overlapping domain decomposition approach to solve the discretized PDE model. In this regard, we parallelized our code by using minimum global communications for solving the stochastic part of the problem. The time discretization results in a system of linear equations which can be solved using efficient iterative solvers. The BiCGSTAB (van der Vorst, 1994) with Jacobi preconditioner is used as the linear solver and the relative tolerance of 10^−6^ is used as the stopping criteria for the linear solver at each step of the ODE time integrator.

Here we propose a novel technique to determine the new timestep during the stochastic opening of many CRUs. Due to the presence of the large numbers of CRUs, the stochastic algorithm that governs the timestep for the next channel transition plays an important role for the computations. As mentioned before, channel state transitions which do not represent openings or closings are, within a given iteration step, independent stochastic events. We can therefore execute several of them within a single iteration. Additionally, typical time steps during an AP are in the range of 0.01 ms, i.e., they are shorter than the diffusion time between neighboring CRUs. Consequently, conductance changing events in different CRUs are statistically independent on the time scale of a single iteration and we can allow for several of them in (distinct) CRUs within one time step.

We introduce two time steps: first the deterministic timestep τ_det_ (which is allowed by the numerical integration of the PDEs) and second the stochastic timestep τ_stoc_. We propose the following algorithm.

**Figure 4:**
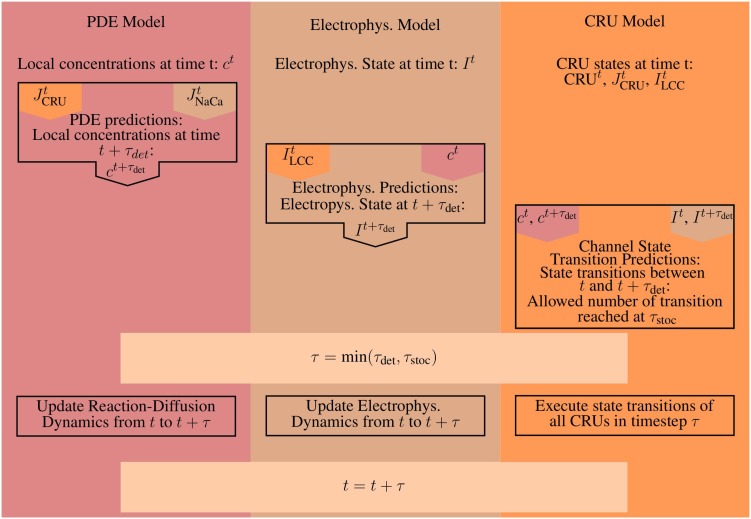
**A schematic illustrating the basic steps for a single iteration**. Orange marks all values produced and all work done by the CRU-Model. The work and results of the PDE-Model are marked in red, everything involved with the Electrophysiology ODE-Model (Electrophys. in the diagram) is marked in brown. A superscript *t* marks the value of a given quantity at time *t*, while a superscript *t* + τ denotes the predicted value of that quantity at time *t* + τ, e.g., the prediction step for the PDE-Model uses the flux values from the CRU and ODE-Model at time *t*. The CRU Transition Prediction step uses both the current values at time *t* and the predicted values at time *t* + τ from the PDE- and ODE-model.

The bulk calcium cycling PDE model and the electrophysiology model are integrated from *t* to *t* + τ_det_, where τ_det_ is the accepted deterministic timestep of the PDE solver. Then, the stochastic channel transitions are predicted from *t* to *t* + τ_det_. Suppose there were *N*_*s*_ conductance changing stochastic events at times *t* + τ_*i*_ where *i* = 1…*N*_*s*_, τ_*i*_ ≤ τ_det_ and 0 ≤ *N*_*s*_ ≤ *N*_*c*_. Here, the time of the stochastic event is τ_*i*_ for the *i*^th^ CRU. In case that there is no stochastic event for a CRU, τ_*i*_ is set to τ_*i*_ = τ_det_. The stochastic timestep τ_stoc_ is determined from the τ_*i*_ as the time by which a maximum number of acceptable transitions is reached. The maximum number has been determined empirically to be sufficiently small with 0.1 *N*_*s*_ to cause no essential difference to simulations with τ_stoc_ sufficiently small to guarantee *N*_*s*_ = 1. Now all the occurring events in the CRUs up to *t* + τ_stoc_ are set to take place at time *t* + τ_stoc_. By doing so, we avoid time steps which are too small for acceptable simulation time. A schematic illustrating a single iteration and how the time steps are determined can be found in Figure 4.

## 3. Results

**Figure 5:**
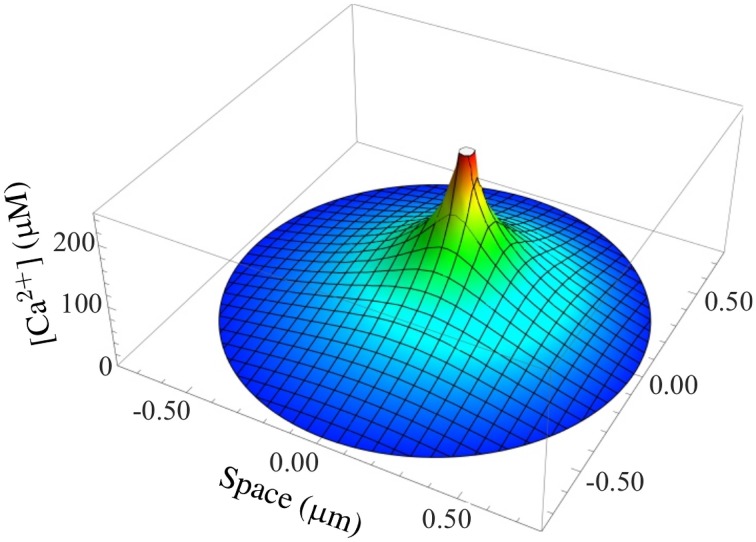
**Simulation of a typical Ca^2+^ gradient in a CRU**. The stationary dyadic [Ca^2+^] profile of a single open RyR is shown.

Our main result is the fully coupled simulation tool. Our motivation was to be able to take stochastic channel state dynamics for each channel in each CRU and the concentration profile within CRUs into account while executing the simulation of (partial) differential equations for other state variables. Figure 5 shows such a concentration profile inside the dyadic space calculated with Equation (17) for a typical RyR current. Gradients are substantial such that distant RyRs and LCCs experience much smaller concentrations and Ca^2+^-dependent transition rates than channels close to an open one.

**Figure 6:**
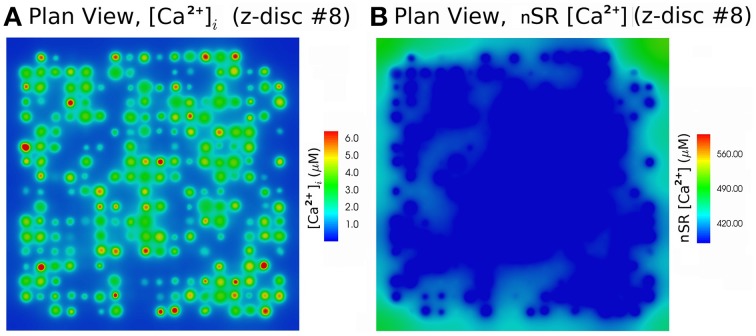
**Free [Ca^2+^]_*i*_ and SR free [Ca^2+^] at 70.0 ms after activation, using the Walker et al. (2014)-RyR-model**. **(A)** Myoplasm [Ca^2+^]_*i*_. **(B)** nSR [Ca^2+^] for the 8*th* z-disc numbered from the bottom in Figure 7. The concentration is color-coded according to the color scale shown. There are 320 CRU per z-disc, with an average of 50 RyR and 12.5 LCC per CRU. See Supplementary Movies 1, 2 for the evolution of [Ca^2+^]_*i*_ and nSR [Ca^2+^] through an AP.

**Figure 7:**
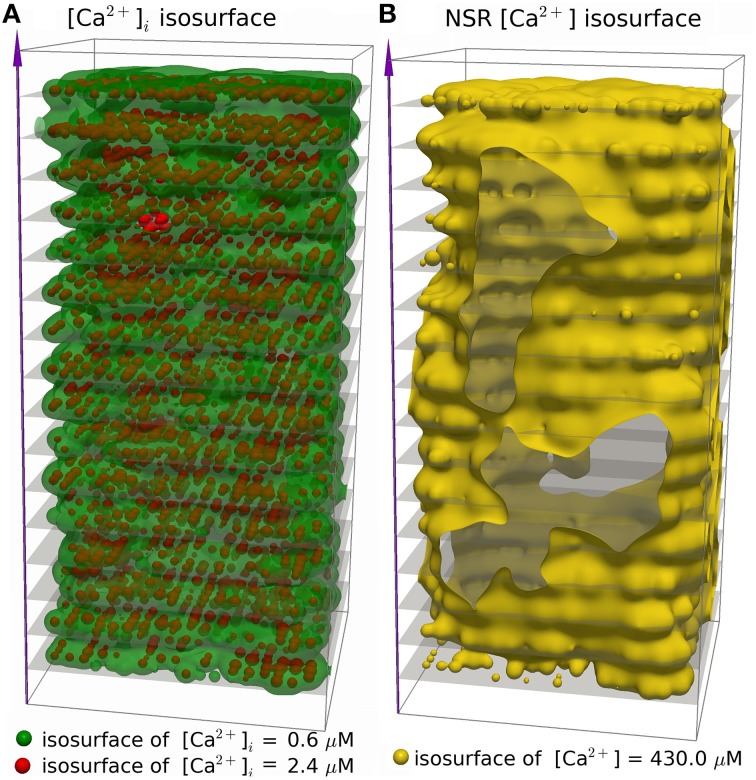
**Three dimensional visualization of spatially resolved [Ca^2+^] at 70.0 ms after activation, using the (Walker et al., 2014)-RyR-model**. **(A)** Isosurfaces show [Ca^2+^]_*i*_ in green for [Ca^2+^]_*i*_ = 0.6 μM and red for [Ca^2+^]_*i*_ = 2.4 μM. **(B)** The yellow isosurface shows free SR-[Ca^2+^] = 430 μM. There are 320 CRU per z-disc, amounting to 5160 CRUs in total, with an average of 50 RyR and 12.5 LCC per CRU. See Supplementary Movies 1, 2 for the evolution of [Ca^2+^]_*i*_ and nSR [Ca^2+^] through an AP.

**Figure 8:**
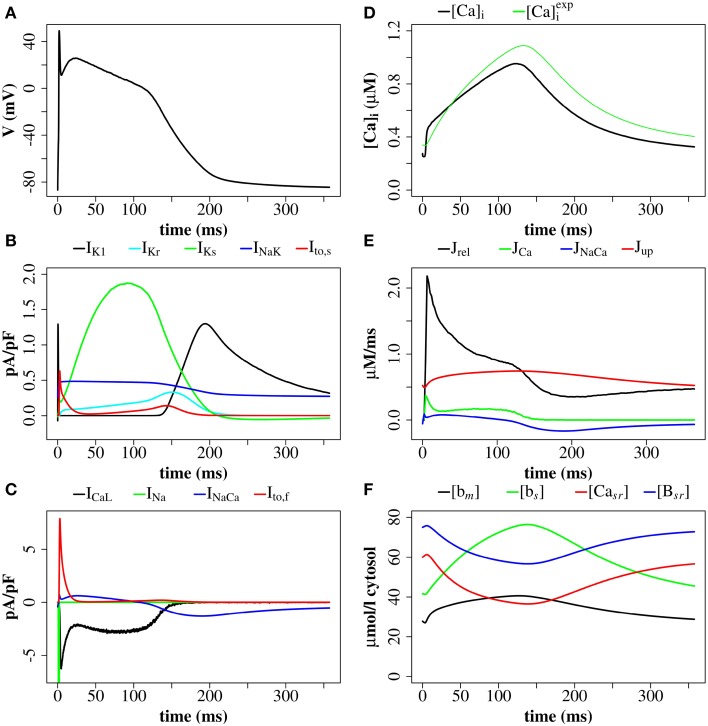
**Membrane potential, ionic currents, and concentrations after a stimulus of 40 mV for 1 ms with the (Walker et al., 2014)-RyR model**. **(A)** Membrane potential. **(B)** Currents I_*K*1_, I_*Kr*_, I_*Ks*_, I_*NaK*_, and I_*to, s*_. **(C)** LCC current (I_*CaL*_), Na^+^/Ca^2+^-exchanger current (I_*NaCa*_), I_*to, s*_, and I_*Na*_ (truncated). **(D)** Average cytosolic [Ca^2+^]_*i*_ and ${\left[Ca^{2+}\right]}_{i}^{exp}$ as defined in Equation (20). **(E)** Ca^2+^ -fluxes: J_*rel*_, J_*Ca*_, J_*NaCa*_, J_*up*_. **(F)** Buffer-bound [Ca^2+^] ([*b*_*m*_], [*b*_*s*_], [*B*_*sr*_]), and nSR lumenal [Ca^2+^] ([Ca_*sr*_]) are plotted. [*b*_*m*_] and [*b*_*s*_] are conventional concentrations in units of μM whereas [Ca_*sr*_] and [*B*_*sr*_] are expressed for simplicity in units of μMν_*sr*_/ν_*cyt*_ (μmol/l cytosol). Plots are of the first AP from a simulation with 16 z-discs.

We simulated 16 z-discs, each with 320 CRUs, and the Ca^2+^ dynamics for this sub-cellular region (~30% of the cardiomyocyte) were coupled to the whole-cell electrophysiology ODE model. It took 64.2 h to solve a single action potential on 848 Intel Xeon E5-2650 v2 2.60 GHz CPUs (central processing units). The Ca^2+^ concentration profile 70.0 ms after stimulus at a single z-disc is shown in Figure 6. The local Ca^2+^ dynamics and corresponding whole cell electrophysiology are shown in Figure 7A and in Supplementary Movie 1. The SR free [Ca^2+^] is visualized in Figure 7B and in Supplementary Movie 2. Currents are shown in Figure 8.

The simulation shows that we can reproduce the whole sequence of Ca^2+^ transients from the initial concentration profile in the dyadic space, via sparks to the whole cell transient. The cytoplasmic [Ca^2+^] is rather heterogeneous, due to randomness of release events as well as variations in CRU size. The concentration of 0.6 μM is reached in almost the whole volume, but 2.4 μM only in the proximity of the the z-discs. The currents reproduce basic features of the Mahajan-model (Mahajan et al., 2008a), and likewise reproduce experimental plots of rabbit Ca^2+^ currents well (Weber et al., 2002), but show some discrepancies mostly related to Na^+^/Ca^2+^-exchanger current.

**Figure 9:**
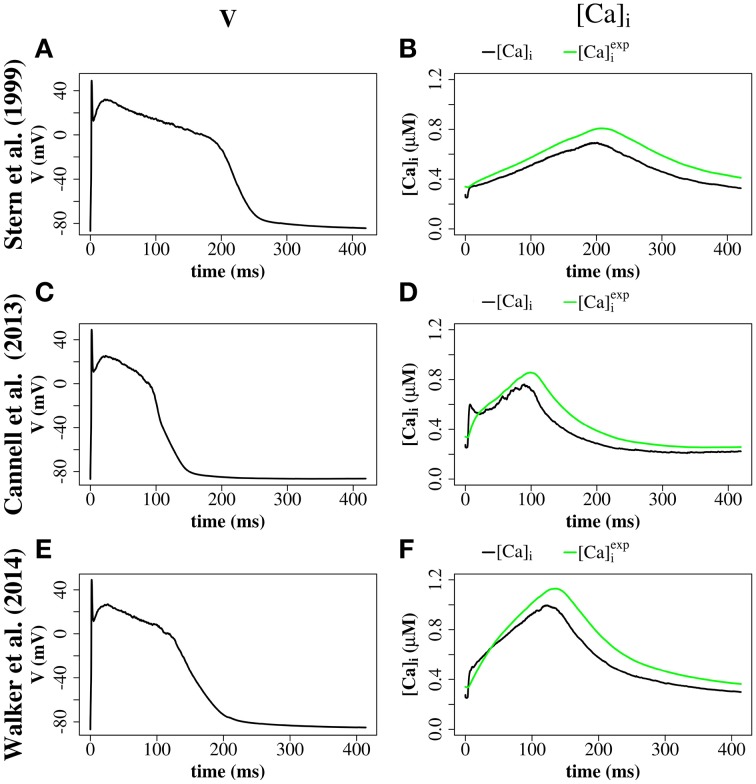
**Comparison of 4-state RyR model (Stern et al., 1999, top panels), 2-state RyR model (Cannell et al., 2013, middle panels), and 2-state RyR model (Walker et al., 2014, bottom panels)**. **(A)** Stern et al. (1999) membrane potential. **(B)** Stern et al. (1999) average cytosolic [Ca^2+^]_*i*_ and ${\left[Ca^{2+}\right]}_{i}^{exp}$ (as defined in Equation 20). **(C)** Cannell et al. (2013) membrane potential. **(D)** Cannell et al. (2013) average cytosolic [Ca^2+^]_*i*_ and ${\left[Ca^{2+}\right]}_{i}^{exp}$. **(E)** Walker et al. (2014) membrane potential. **(F)** Walker et al. (2014) average cytosolic [Ca^2+^]_*i*_ and ${\left[Ca^{2+}\right]}_{i}^{exp}$. Plots are of the first AP from a simulation of a single z-disc.

**Figure 10:**
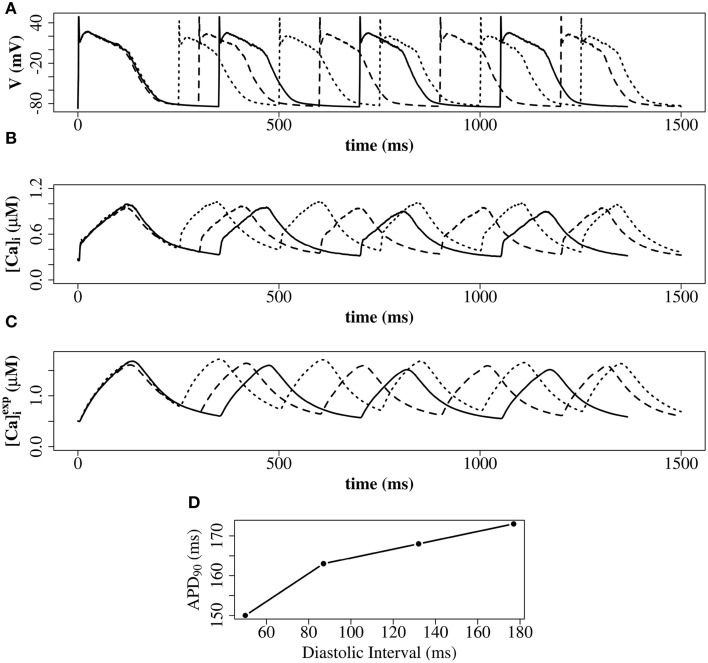
**Restitution properties with the (Walker et al., 2014)-RyR model**. **(A)** Membrane potential. **(B)** Average cytosolic [Ca^2+^]_*i*_]. **(C)** Average cytosolic ${\left[Ca^{2+}\right]}_{i}^{exp}$ as defined in Equation (20). Solid line: basic cycle length (BCL) = 350 ms; dashed line: BCL = 300 ms; dotted line: BCL = 250 ms. At cycle length 350 ms the DI was 177 ms. At cycle length 300 ms the DI was 132 ms. At cycle length 225 ms the DI was 87 ms. **(D)** Constant BCL Restitution Curve (Otani and Gilmour, 1997). For clarity, the AP with the shortest DI in **(D)** is not shown in **(A–C)**. These simulations have been carried out with a single z-disc.

We explored the use of three RyR models which all produce realistic action potentials (Figure 9). When using the (Stern et al., 1999) model there were slow kinetics of Ca^2+^ release and a low RyR maximum open percentage (2.7%). Gain (the ratio of *J*_*rel*_ to *J*_*Ca*_) was ~4 during the first 50 ms of the AP and ~3 for the remainder of the AP (AP duration = 265 ms, basic cycle length BCL = 350 ms). With the (Cannell et al., 2013) model many RyRs open and close (spark-like) within 20 ms of the start of the AP, and gain was high in this early phase of the AP (~10) and moderate in the later AP (~6) and there was a corresponding fast rise in the [Ca^2+^]*i* with an early peak at ~10 ms, followed by a reduction of [Ca^2+^]_*i*_ and a second [Ca^2+^]_*i*_ rise and fall through the AP. With the (Walker et al., 2014) model gain was ~6 in the early AP, then ~2 in the remainder of the AP, and there was a relatively high RyR maximum open percentage (32%). The (Cannell et al., 2013; Walker et al., 2014) 2-state RyR-schemes have markedly faster (and more physiological) kinetics of Ca^2+^ release than the (Stern et al., 1999) 4-state scheme. With the (Walker et al., 2014)-RyR scheme our model displays expected restitution properties with shorter APD and [Ca^2+^]_*i*_ transient with shorter BCL (Figure 10).

## 4. Discussion

This proof of concept study demonstrates a new multi-scale model of CICR linking three spatial scales: (1) detailed molecular stochastic modeling of the CRU at a continuous spatial scale; (2) a whole-cell ODE electrophysiology model, which describes the potassium channels, the voltage gated sodium channels, the sodium-potassium pump, and integrates all membrane fluxes to derive the total membrane current and voltage; (3) a PDE FEM calcium diffusion model representing myoplasmic and nSR Ca^2+^ diffusion between CRUs and between z-discs. One rationale for this approach is that it allows the removal of the artificial compartment for the sub-membrane space, and hence provides a more quantitative modeling of diffusion processes and gradients.

Taking gradients in the dyadic cleft into account in a simulation with thousands of CRUs would be impossible without the use of the stationary Green function. An estimate of the advantage of the quasi-static approximation with respect to computational speed can be obtained from estimating the number of operations per cleft. Our approach requires to solve a linear system of equations whenever channels open or close or the boundary condition or the jSR concentration has changed significantly. That means ${\left(N_{RyR}+N_{LCC}\right)}^{2}$ operations each. Simulating the concentrations inside the cleft would require about 1000 grid points and a time step in the microsecond range (see Thul and Falcke, 2004). Consequently, it would be at least a few hundred times slower than the use of the stationary Green function. Applying the dynamic Green function would entail similar computational efforts, since it converges extremely slowly (Bentele and Falcke, 2007).

To our knowledge, our model is the only whole cell AP model which includes CRUs clustered around the z-discs (a proxy for explicit T-tubules) *and* spatial resolution of intra-CRU [Ca^2+^] with realistic steep [Ca^2+^] gradients. Other approaches include the multiscale model of Restrepo and Karma (2009) which simulates 20,000 CRUs with Markov models for individual LC- and RyR-channels in a three-dimensional grid interacting with a membrane voltage ODE-model (Mahajan et al., 2008a). This was developed further by Nivala et al. (2012) and was modified to a bidomain approach with finite elements belonging to cytoplasm or SR. A related model of Williams et al. (2011) incorporates energetic coupling of the RyRs, and non-junctional RyRs, but is used only for the analysis of the diastolic RyR dynamics without an ODE electrophysiology model. Walker et al. (2014), based on Williams et al. (2011), developed a finite volume model of a single CRU which includes modeling of intra-CRU calcium concentration differences, diffusion of Ca^2+^ and mobile buffers, T-tubules, Markov chain models for the channels and excitation-contraction coupling gain. Cannell et al. (2013) investigated spark dynamics and termination using a two-state RyR model with modeling of intra-CRU calcium concentration differences, but until our current study the Cannell et al. (2013) model had not been coupled to a whole cell geometry or to an action potential model. Torres et al. (2014) modeled a modified local control mechanism to simulate confocal image data and to explore the relationship between Ca^2+^ transients and the activation of non-junctional RyR clusters of myocytes with sparse T-systems. A finite-element model has been implemented by Hatano et al. (2011) which incorporates subcellular structures including the T-tubule system, SR, myofibrils and mitchondria, where electrophysiology and local calcium dynamics are coupled to a local myofibril contraction model to simulate local and global contraction. They model the spatial detail of CICR release and local contraction for three myofibrils of one sarcomere length but do not model the CRU [Ca^2+^] in spatially resolved detail.

We explored the use of three RyR models: the 4-state model of Stern et al. (1999), the 2-State induction decay model of Cannell et al. (2013), and the 2-State model of Walker et al. (2014) [similar to the Cannell et al., 2013-model except with the addition of modulation of the RyR open probability by junctional RyR-lumenal [Ca^2+^] ($c_{jsr}^{i}$)]. With the (Stern et al., 1999) RyR-model peak [Ca^2+^]_*i*_ equals 0.8 μM with time to peak of 200 ms. With the (Cannell et al., 2013) model, dual peaks in [Ca^2+^]_*i*_ were observed with an early peak of [Ca^2+^]_*i*_ = 0.6 μM at time to peak ~10 ms, with the principal peak [Ca^2+^]_*i*_ transient of ~0.8 μM and time to peak 100 ms. With the (Walker et al., 2014) peak [Ca^2+^]_*i*_ was ~1.0 μM and time to peak 130 ms. The time to peak [Ca^2+^]_*i*_ for both the 2-state models is comparable to that reported for the rabbit by Weber et al. (2002). The early [Ca^2+^]_*i*_ local maximum with the (Cannell et al., 2013) model may be explained from the origins of the (Cannell et al., 2013) model, which is from fits to experimental data from Ca^2+^ sparks and blinks, and has never been previously used in a model of prolonged CICR resulting from an AP. Indeed the authors of this model state that the model equations are not designed to capture behavior at resting [Ca^2+^]_*i*_ with fully loaded SR. The RyR-dynamics of this model with its current parameters are therefore reproducing spark like behavior in an AP. We had to introduce strong buffering inside the dyadic space and jSR-depletion in order to reach closing rates sufficiently fast for termination of release at the end of an action potential. However, this proof of concept investigation does not provide evidence that induction decay alone is an insufficient mechanism to cause the termination of CICR in an AP. Rather, the model provides a platform for investigating AP CICR. Careful experimentally led tuning of the 2-state model parameters alongside Ca^2+^ buffering parameters and the jSR refill flux will allow assessment of the feasibility of induction decay as the sole CICR termination mechanism in an AP. A second possible explanation for the early local maximum in [Ca^2+^]_*i*_ with the (Cannell et al., 2013)-model is that in our model we do not explicitly represent the [Ca^2+^]_*i*_ uptake and release by the mitochondria. Although mitochondrial [Ca^2+^]_*i*_ uptake and release is controversial on such fast timescales (Boyman et al., 2014), there is evidence that fast early mitochondrial [Ca^2+^]_*i*_ uptake can act like a fast stationary buffer, and that inhibition of fast uptake through a specific inhibitor of the mitochondrial Ca^2+^ uniporter (MCU) can result in an early local maximum of the [Ca^2+^]_*i*_ transient (Maack et al., 2006). Furthermore, the data in Figure 9 show that the [Ca^2+^]_*i*_ recorded in experiments via a fluorescent Ca^2+^ probe such as Fluo-4 [approximated as (${\left[Ca^{2+}\right]}_{i}^{exp}$), as defined in Equation 20] would smooth-out any early [Ca^2+^]_*i*_ peak, if this were a genuine experimental feature of cardiomyocyte CICR. In AP simulations with the 2-state RyR models, some local high calcium transients at CRU-sites remained into the diastolic period, associated with CRUs where some RyRs remained open. A similar phenomenon has been described in the setting of spontaneous Ca^2+^-sparks by Stern et al. (2013), who used an RyR-scheme similar to Walker et al. (2014) [a 2-state model, with modulation of the open probability by junctional RyR-lumenal [Ca^2+^] ($c_{jsr}^{i}$)].

The discrepancies between our simulations and the ODE-Mahajan model with respect to Na^+^/Ca^2+^-exchanger current illustrate the value of spatially resolved modeling in exploring detailed properties of Ca^2+^ dynamics. Using the same Na^+^/Ca^2+^-exchanger model as Mahajan et al. (2008a) we were not able to reproduce the same time course of the total Na^+^/Ca^2+^-exchanger current. In both models the current has the same general profile, with a short period in reverse mode (positive current) in the first phase of an action potential, followed by forward mode (negative current) during the *V*-plateau and decline. However, in our approach the *I*_*NaCa*_ current turns negative much later during the plateau than in the ODE model. The Na^+^/Ca^2+^-exchanger depends on the [Ca^2+^] in sub-membrane space in the ODE model and on the cytoplasmic concentration *c* in our simulations, including of course the regions with high *c*-values at and close to CRUs. The main reason for the differences is that the sub-membrane [Ca^2+^] in the ODE model exhibits a sharp rise and decline while the average myoplasmic concentration shows slower dynamics. We have experimented with localizing the Na^+^/Ca^2+^-exchanger molecule density in proximity to the CRUs to reflect the observed higher abundance of Na^+^/Ca^2+^-exchanger near the CRU (Scriven et al., 2000, 2002; Jayasinghe et al., 2009; Scriven and Moore, 2013). However, that has not fully resolved the discrepancies with respect to the ODE results yet. We expect to gain detailed insights into the factors shaping the Na^+^/Ca^2+^-exchanger current through investigation of these problems.

## 5. Conclusions and limitations

We showed on a level of proof-of-concept that multiscale modeling of cardiomyocyte ECC from sub-dyadic scales to many z-discs using full partial differential equations is possible. We demonstrate that the model produces realistic physiology on these scales and has the potential to provide new insight into subcellular mechanisms and structures. Maybe the most severe limitation of this modeling approach is the requirement for high performance computing (HPC) to run it. We expect some improvement of simulation efficiency by more specifically tailored numerical methods, however the requirement for HPC will remain. The use of the Green function inside the dyadic cleft requires linearity of the reaction diffusion equations there. That excludes non-linear buffering terms and allows for linear buffering only. Some of the limitations of our model arise from its early state of development and will be removed with inclusion of more detail like e.g., anisotropic diffusion, mitochondria, more detailed buffering, and non-junctional RyR. We used modular programming as far as possible to ease the exploration of a variety of ion channel models and other species specific membrane potential dynamics.

## Author contributions

NC implemented the PDE model and coupled this with the CRU model and cell electrophysiology ODE model. JV co-implemented the cell electrophysiology ODE and PDE model. WN and SG implemented the RyR Markov-models. WN implemented the CRU model and co-implemented the PDE model. NC, WN, JV, SG carried out simulations, processed data from simulations and produced results figures. SG, NC, WN, JV, MF drafted the manuscript. MF conceived the CRU model, the PDE model and the coupling of the model scales. All authors approved and assisted in redrafting the final manuscript.

## Funding

This study has been supported by DFG grant FA350/9-1, DFG GRK 1772, BMBF grant eMed:SMART and a collaborative grant from the German Centre for Cardiovascular Research to MF and S. Luther, MPI DS Göttingen.

### Conflict of interest statement

The authors declare that the research was conducted in the absence of any commercial or financial relationships that could be construed as a potential conflict of interest.

## Footnotes

^1^A factor of *h* on the rhs of Equation (15) cancels with the same factor in the denominator of Equations (18, 19) turning point sources into line densities.

## Supplementary material

Supplementary Movie 1 **Simulation of the first AP with 16 z-discs using the (Walker et al., 2014)-RyR model. (A)** Shows the three-dimensional 16 z-disc myoplasmic [Ca^2+^] by means of 0.6 μM and 2.4 μM free myoplasmic [Ca^2+^]_*i*_ isosurfaces; **(B)** shows the membrane-potential and the mean myoplasmic [Ca^2+^]_*i*_ transient; **(C)** is a section through the PDE solution at the 8*th* z-disc from the top with the image colored by free myoplasmic [Ca^2+^].

Supplementary Movie 2 **Simulation of the first AP with 16 z-discs using the (Walker et al., 2014)-RyR model. (A)** Shows the three-dimensional 16 z-disc [Ca_*sr*_] by means of a 430 μM isosurface; **(B)** shows the membrane-potential and the mean myoplasmic [Ca^2+^]_*i*_ transient; **(C)** is a section through the PDE solution at the 8*th* z-disc from the top with the image colored by [Ca_*sr*_].
